# A Dynamic Representation Solution for Machine Learning-Aided Performance Technology

**DOI:** 10.3389/frai.2020.00029

**Published:** 2020-05-08

**Authors:** Jason Palamara, W. Scott Deal

**Affiliations:** ^1^Department of Music and Arts Technology, Indiana University-Purdue University Indianapolis, Indianapolis, IN, United States; ^2^Donald Tavel Arts and Technology Research Center, Department of Music and Arts Technology, Indiana University-Purdue University Indianapolis, Indianapolis, IN, United States

**Keywords:** music and machine learning, music and AI, dynamic representation, machine learning aided performance, improvisation, Ableton Live, Max for Live, music technology

## Abstract

This paper illuminates some root causes of confusion about dynamic representation in music technology and introduces a system that addresses this problem to provide context-dependent dynamics for machine learning-aided performance. While terms used for dynamic representations like forte and mezzo-forte have been extant for centuries, the canon gives us no straight answer on how these terms must be applied to literal decibel ranges. The common conception that dynamic terms should be understood as context-dependent is ubiquitous and reasonably simple for most human musicians to grasp. This logic breaks down when applied to digital music technologies. At a fundamental level, these technologies define all musical parameters using discrete numbers, rather than with continuous data, making it impossible for these technologies to make context-dependent decisions. The authors give examples in which this lack of contextual inputs in music technology often leads musicians, composers, and producers to ignore dynamics altogether as a concern in their given practice. The authors then present a system that uses an adaptive process to maximize its ability to hear relevant audio events, and which establishes its own definition for context-dependent dynamics for situations involving music technologies. The authors also describe a generative program that uses these context-dependent dynamic systems in conjunction with a Markov model culled from a living performer–composer as a choice engine for new music improvisations.

## Introduction

As of this writing, music technologies (software and hardware) cannot perform relative dynamics, only absolute dynamics. If a given system is set to play a tone at 0 dBFS, it will do so regardless of context. Music technologists of many stripes, such as professional audio engineers, often adapt methods for handling this, for instance, how professional audio engineers use the faders on a mixer to adapt the incoming audio signals for a particular situation, given the particulars of the room, the number of people present, and many other factors. However, music technologies do not adapt themselves to different contexts natively, which often causes amateur or nascent users to make mistakes leading to many amplitude-related errors, such as feedback or the tendency to mix without dynamic contrast (“brickwalling”) (Devine, 2013).

Confusingly, there exists a dizzying preponderance of methods music technologists use to represent dynamic levels (dB Full Scale, dB Sound Pressure Level, MIDI velocities, to name a few) (Dannenberg, 1993). These various systems, helpful as they are in many respects, thus inveigle one of the four fundamental properties of musical sound (volume, pitch, duration, and timbre) in a haze of pseudo-scientific mystery. The recent development of the LUFS and LKFS (the EBU R128 Standards, released in 2014) scales may do much to alleviate the preponderance of complaints against the rising levels of loudness (where the amplitude level of one piece of music is compared to the next), but this scale will not fix a lack dynamic contrast within a piece of music as it is being composed, mixed, or performed via improvisational or generative technologies.

One might think that the system of dynamic representation that has been with us for centuries would have been definitively codified long ago, but as illuminated by Blake Patterson, many musicians don't follow a composer's intent when they play to any appreciable degree (Patterson, 1974). Moreover, much anecdotal evidence suggests that this unfamiliarity with the various systems for representing dynamic levels may result in a general dismissal of the importance of one of the four fundamental parameters of sound. As noted by Kyle Devine in his article Imperfect Sound Forever: loudness wars, listening formations and the history of sound reproduction, quite often, the lack of dynamics in modern music has more to do with market-driven forces and personal taste than with user's technological naiveté (Devine, 2013).

Matthias Thiemel goes to admirable length to explain that, concerning acoustic music makers, dynamics have always been a fundamental parameter with which musicians “create meaning and structure” (Thiemel, 2001). However, he also goes to great length to explain that the history of the concept in music is one that eschews a literal understanding of exact loudness levels, in favor of an ever-adapting definition, which must change according to the whims and predilections of the time in which a composition is conceived. Thus, as he explains, the dynamic fortissimo might mean one thing in an early piece of Beethoven but means something completely different in a later piece by the same composer. That dynamics must be understood in the context of the composition and composer who wrote them is largely understood by professional performing musicians and musicologists, and this definition changes not only from instrument to instrument, from piece to piece, and even depends on the range of the instrument in question or the venue in which the dynamic in question is to be played.

A professional trumpet player, upon encountering the dynamic forte in the midst of an opera score of Puccini, may be capable of calculating the required dynamic using some internal concatenation of variables including the composer's intention, the conductor's most recent indication, the range of the given note, the size of the hall, and the probability of accidentally overpowering the ensemble even though no “solo” was called for in the score. The number of variables occurring to the player in question will vary greatly due to a great many factors, i.e., the maturity and experience level of the player, the cultural setting, however, perhaps this short list will hopefully illustrate the sheer number of factors involved in such a decision, which in this case might result in the player in question playing the aforementioned note with a measurable dynamic level of 82 dB. In another setting, the same player might play the same excerpt at 65 or 90 dB. Dynamics would seem to be “all relative.”

For music technology, however, this malleable understanding of musical dynamics presents a sizable problem. For instance, if one was tasked with transcribing the composer's handwritten score into one of the many notation programs currently available (Finale, in this case), the system would automatically assume that the forte marking in question corresponds to a certain MIDI velocity, which, when played via a digital instrument, will have the same results every time the user hits the space bar. In the case of Finale, a dynamic marking of forte corresponds to a MIDI velocity of 88 (out of 128 possible values, from 0 to 127). When this velocity level reaches the digital instrument, the dynamic will be converted into a loudness level, which is easily definable as 88/128ths of the instrument's total volume. Every time this instrument plays this dynamic level, the same volume level will be called upon to playback, no matter the context. If a composer wants to prepare a “fixed media” score or part for the aforementioned trumpet player, either there will need to be another performer who will manage dynamics for the fixed media part to provide context-appropriate dynamic choices or the composer will tend to avoid large dynamic contrasts altogether.

## Method

**Figure 1:**
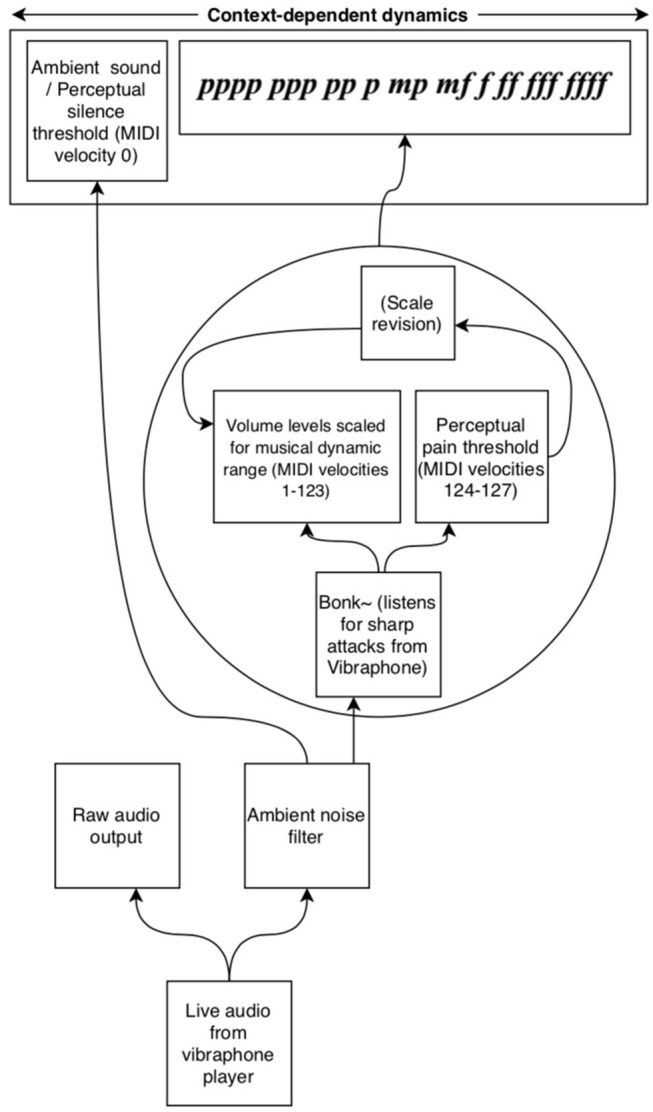
A schematic of Avatar's process for establishing context-dependent dynamics by listening to incoming audio.

**Figure 2:**
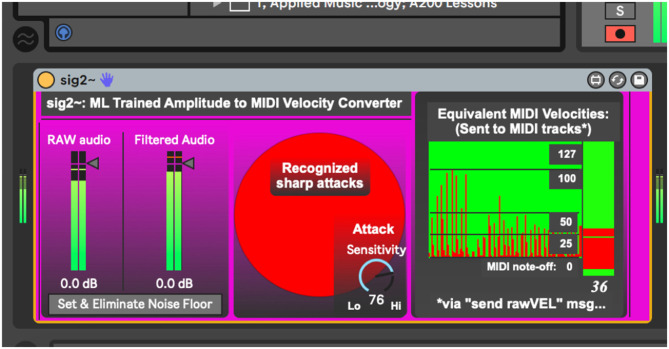
Sig2~.amxd, a program that uses machine learning to establish a conceptual framework for silence and the pain threshold.

**Figure 3:**
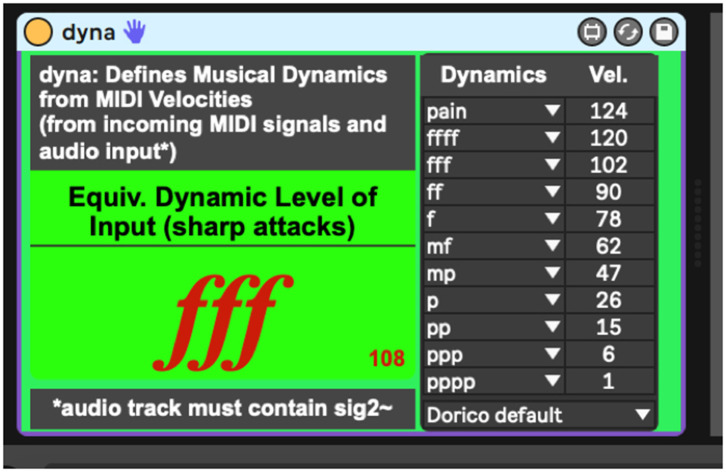
Dyna.amxd, a program that translates MIDI velocities into various dynamic representation systems.

To address the issues above, the authors here present a network of interconnected programs, collectively called *Avatar*, which may begin to fill the gap between musical technologies and context-dependent musical dynamics (Figure 1). The system has been designed trained to listen for a specific timbre (the vibraphone), filtering out noise, and non-intentional sound. This system then uses incoming amplitude levels to establish an adaptive perceptual framework for two key musical perception concepts, silence and the pain threshold. Finally, the system provides context-dependent MIDI velocities and musical dynamic representations of the audio it is hearing. These dynamics can then be used by generative music systems to play along and inform musical choices with context-dependent volume levels. In performance, this system follows the dynamics of the human vibraphone player, as a human collaborator would. The current context-dependent dynamic system is composed of two programs, *sig2*~ (Figure 2), and *dyna* (Figure 3), and has been written in the Max-for-Live language, to facilitate use by music technologists in live performance using Ableton Live.

**Figure 4:**
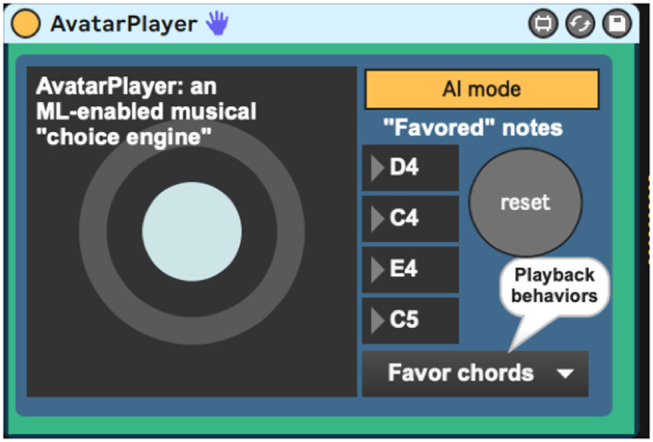
AvatarPlayer.amxd, a program that uses machine learning to generate new musical choices in a given player's style.

**Figure 5:**
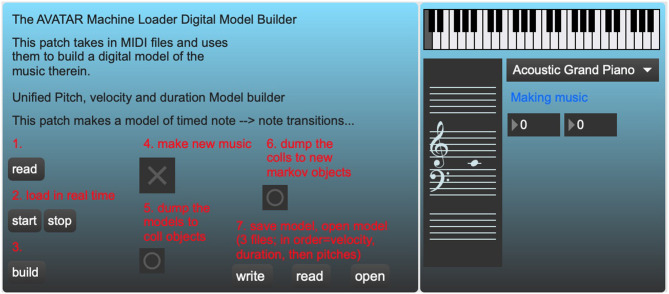
AvatarMachineLoader.maxpat, a standalone Max program that builds a Markov model of pitch transitions from MIDI files.

A third program, the *AvatarPlayer* (Figure 4), which will be discussed toward the end of this section, makes use of a pitch transition Markov model, culled from performances by a living composer–improviser. This program takes in messages from *sig2*~ and *dyna* to generate context-dependent musical choices as it plays along with live vibraphone input. A fourth program, the *AvatarMachineLoader* (Figure 5), has been developed and used by the authors to create a database of Markov transitions that are used by the *AvatarPlayer* to generate new music.

Aside from the standard objects found natively in the Max language, these programs also make use of a number of external (added) Max objects. Most notably among these are a number of external Max patches developed by the authors with the prefix *HIMI* (Human Inclusive Musical Intelligence, a tangentially related project), the *bonk*~ object (from Puckette, Apel, and Zicarelli, as revised by Böhm), the *ml.markov* object (from Benjamin Smith's *ml*.^*^ machine learning for Max external package; Smith and Deal, 2014), and a number of utility objects from Karlheinze Essl's RTC-lib package of externals (Essl, 1988). With the exception of the objects from the *HIMI* library, which will be included in the commercial release, these external objects are open source or commonly available via Cycling “74”s Max Package manager. To produce MIDI files from audio recordings, the authors also use the *Onsets and Frames* audio-to-MIDI converter from Google Magenta (Hawthorne et al., 2017; Dinculescu et al., 2019), which may be implemented via JavaScript, Python, or used online, and Ableton Live's three built-in audio-to-MIDI converters.

## The Importance of Perceptual Frameworks, Silence, and Pain

The system we present begins by establishing some fundamental perceptual frameworks extant within a human's musical understanding (Buettner, 1981), but which have been largely absent from the world of musical technologies. Though John Cage is correct in asserting that there is “no such thing as silence” (Cage, 1992), as argued by Elizabeth Hellmuth Margulis, the perception of silence is fundamental to our appreciation of music in general (Margulis, 2007). Cage's arguments aside, human beings appreciate music not by taking in a stream of audio and giving attention to the loudest elements, but by framing music as what happens between a conceptual understanding of silence (here defined as ambient sounds, incidental noises, and unintentional sound, which does not pertain to the music presented) and sounds that occur beyond a loosely defined perceptual pain threshold. While the technical human pain threshold corresponds to volume levels over 120 dB SPL, many listeners establish a more personal definition that most likely includes any intensity over 90 dB SPL in most contexts (Smith, 1970). As Margulis states, silences “facilitate processing by chunking the [musical] stream into units whose elements pertain to one another and should be understood, evaluated, and remembered together, by allowing time for the listener to synthesize and reflect on the chunk that has just passed” (Margulis, p. 5). Conversely, the perceived pain threshold provokes the listener to avoidance, covering, or protecting their ears, and in extreme cases, leaving a venue while the music is still happening. These concepts are the fundamental boundaries of human music-making, beyond which a musical performance is apprehended as “too quiet,” “too loud,” or “painful” (Fisher, 1929). For music technologies, without an understanding of these two fundamental concepts, there can be no context-dependent musical dynamics (Cope, 2004; Collins, 2012).

## The *sig2~* Program

The *sig2*~ program, the first link in the context-dependent dynamic system, begins by taking in raw audio, measuring the maximum and minimum levels of audio levels it encounters. In human musical performance, these definitions change over time, and so *sig2*~ changes, accordingly, adapting its minimum (silence, noise floor), and maximum (pain threshold) throughout the performance as a human listener might.

**Figure 6:**
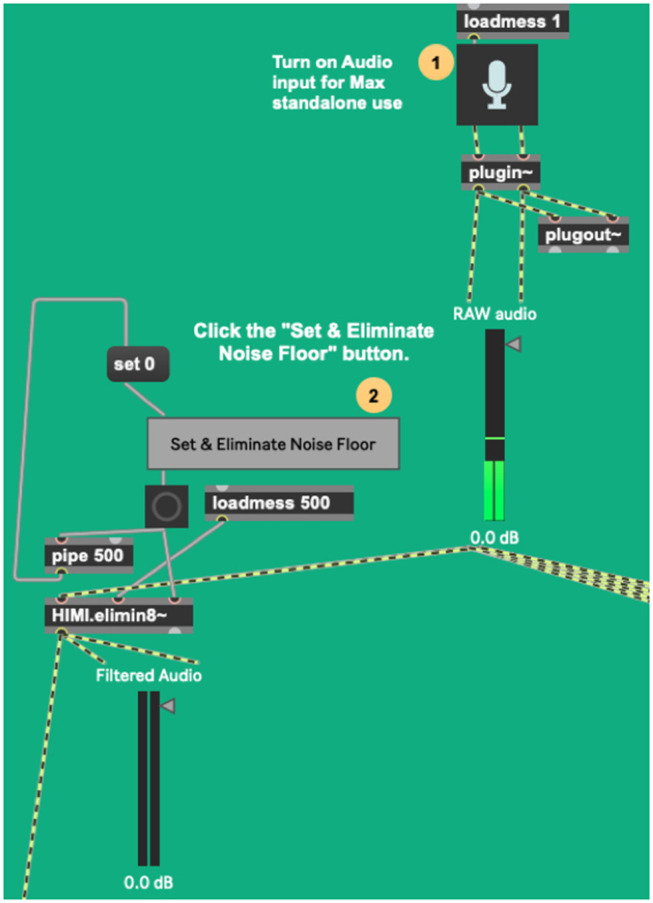
The process beneath sig2~'s noise floor elimination apparatus.

**Figure 7:**
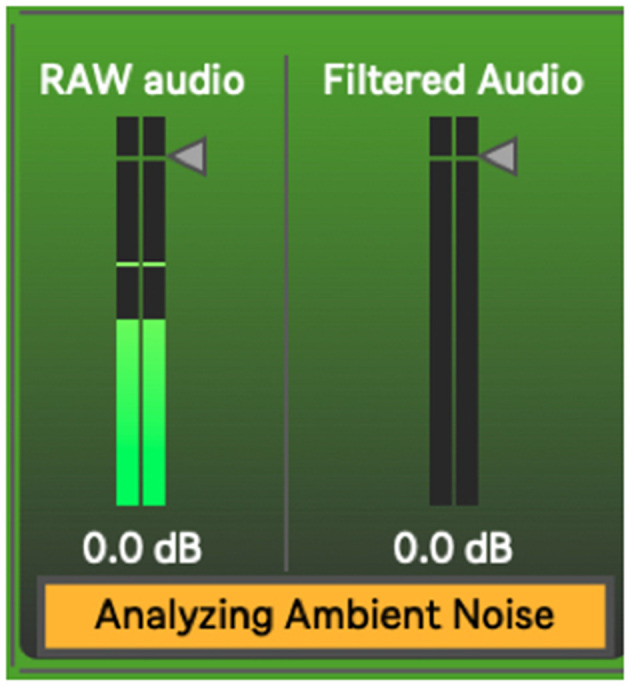
Sig2~'s noise floor elimination apparatus at work.

The *HIMI.elimin8*~ object (Figure 6), developed by the authors for a tangentially related project, is an adaptive filter algorithm that controls a noise gate, which is here useful for filtering ambient sound out of the incoming audio. It takes incoming audio for a short period of time (500 ms), measures its average strength, and gates the incoming signal accordingly, passing only signals stronger than the established noise bed through (Figure 7). This process begins automatically once the device is loaded but can be manually reset if need be. As a human listener ignores the ambient hum of an air conditioning unit while trying to listen to live music, this system works best if the performer remains as quiet as possible during this setup process so a noise bed definition can be made. After establishing this noise bed, the system is optimized to listen only for strong signals and establish its dynamics with anything below this noise bed defined as a non-musical event. As with any gating process, there is a danger here that if the gate is set too high, it will cut out audio intended for the performance. If this occurs, the user has two options, increase the signal (feed the system a higher level of gain), or simply hit the “Set & Eliminate Noise Floor” button again.

**Figure 8:**
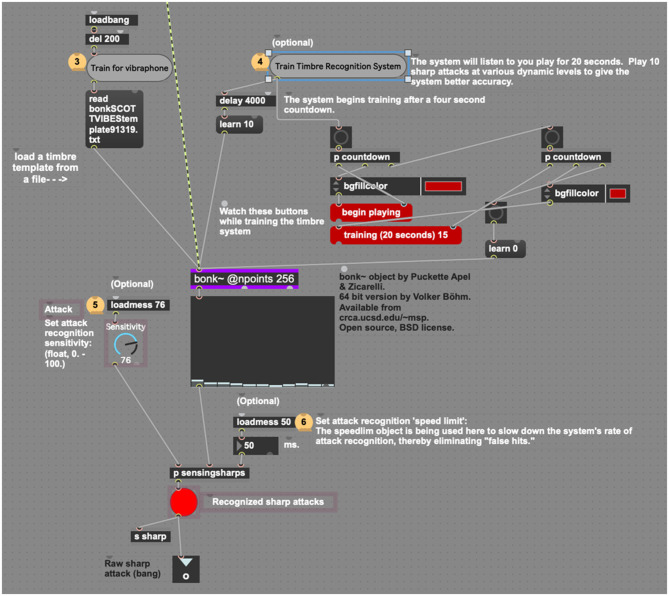
The timbre recognition system, using bonk~.

When using reactive electronics in a live audio situation it is greatly advantageous to filter unwanted sounds, sustaining resonances, and incidental sounds so the system may listen specifically for the performer using the system, rather than trying to distinguish the performer's sound from an undifferentiated stream of audio. Thus, *sig2*~ uses *bonk*~, a common external Max object that may be trained to listen for specific timbres, to build a spectral template of the player's audio (*bonk*~ was developed by Miller Puckette, Theodore Apel, and David Zicarelli, 64-bit version by Volker Böhm) (Puckette et al., 1998). This template can be saved or rewritten if the player is going to use the system again later. By clicking the “Train Timbre Recognition System” button (Figure 8), the user enables the *bonk*~ object to listen for the timbre of the instrument you are playing. As the current project uses a vibraphone player as the listening target, the authors have created a timbre template for vibraphone, which is automatically read by *bonk*~ upon loading the device. This system will operate with no training data but will simply listen for any incoming sharp attack rather than distinguishing a specific instrument from incidental noise.

Since *bonk*~ parses an incoming audio stream into 11 frequency bands, any of which may recognize a frequency extant in the timbre it is hearing, it was useful to include a dial control that sets attack sensitivity (0–100). After repeated trials, it was discovered that *bonk*~ best recognizes the timbre of the vibraphone above a sensitivity setting of 76. Similar trials would likely be necessary to train and perfect the process for another instrument's timbre. Another purpose behind using the *bonk*~ object is to identify sharp attacks and pass them on to the *dyna* device. Upon detecting a sharp attack, *sig2*~ sends a message *wirelessly* to any related objects in the session via the “sharp” message.

The attack recognition “speed limit” default of 50 ms slows there cognition process to within human performance limits. As of this writing, few human musicians can play notes at a rate faster than 10 Hz (Martin and Martin, 1973). Jason Barnes, the world's first true cyborg drummer, when wearing his robotic drummer prosthesis, can play at speeds up to 25 ms, hence the default speed limit caps the system's use to provide for less glitchy playback (Weinberg and Driscoll, 2006). At this point, human perception comes into play as well, as, beyond 20 ms, human beings find it hard to distinguish beats as distinct events, rather, perceiving them as connected events or waves.

*Sig2*~ uses another adaptive algorithm to establish a perceived pain threshold. Upon encountering strong audio levels, *sig2*~ again lowers its sensitivity using the *HIMI.peakamp*~ object. This process is rather simple but continually calculates an overall maximum peak amplitude from the audio it has encountered. *Sig2*~ then uses this maximum to scale incoming audio peaks to a MIDI-friendly 128 integer range (0–127). In this manner, if the system suddenly encounters a greater amplitude level than previously encountered, it simply adapts its scale to accommodate the new peak. The newly defined 128 integer MIDI velocity is sent out of the device via the output at the bottom and also “wirelessly” via the “rawVEL” send object. By establishing these perceptual frameworks and maintaining in real time, the *sig2*~ program can be relied on to convert incoming audio levels into MIDI velocities, giving any connected program a reliable dynamic context in an easy-to-use MIDI format.

## The *Dyna* Program

The *dyna* program is the second device that makes up the context-dependent dynamic system. *dyna* takes in the equivalent MIDI velocities sent from the *sig2*~ device (via the “*rawVEL*” message) and defines dynamic ranges either derived from various popular music notation programs or as defined by the user. The sharp message, also sent by *sig2*~ acts as a trigger for the dynamic representations, giving the performer a context-dependent dynamic of their volume level as they play.

**Figure 9:**
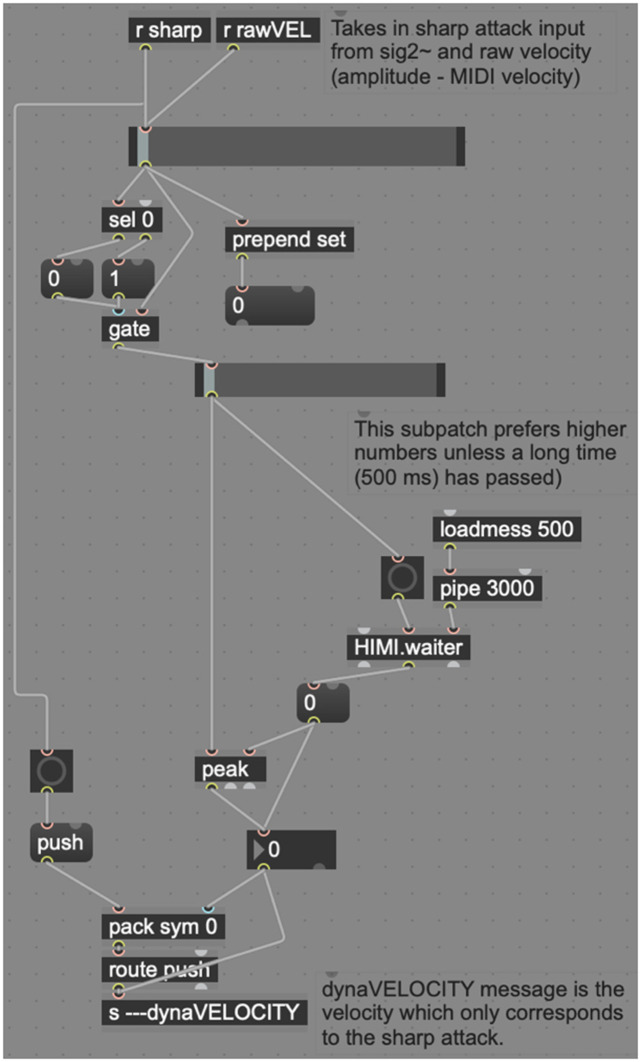
Dyna's sharpvelocitycalculation patch.

Inside the “sharpvelocitycalculation” subpatcher (Figure 9), *dyna* combines the incoming “sharp” message (indicating that a sharp attack has occurred) and the raw MIDI velocity of the most recent attack into a single message. One of the stickiest problem *dyna* addresses is the visual feedback to the performer, which needs to be extremely responsive (new higher dynamics may happen very quickly) but slow enough that the visual feedback system does not constantly “twitch” from dynamic to dynamic quicker than can be read by a human. To counteract this problem, this subpatcher makes use of another preexisting object called *HIMI.waiter*, a Schmitt–Trigger delay that waits until all inputs have ceased before beginning a short delay and then sending a second bang. In the interim, if *HIMI.waiter* receives further input, the delay is canceled, and the process is reset. *HIMI.waiter* slows this process and allows the system to give preference to louder dynamics and ignore quick reflections at lower dynamics. This is modeled after human perception, which prefers louder dynamics, instead of perceiving quick reflections as reverberation (Doyle, 2004).

The actual dynamic definitions further illuminate the problems inherent in the dynamic representation used by many of today's most popular music software. As shown in Table 1, there appears little agreement regarding dynamic and their corresponding MIDI velocities among programs such as Finale, Sibelius, MuseScore, and Dorico. Thus, with dyna, the authors have picked what seems to be the best solution to MIDI velocity dynamics currently on the market and also provided options for users to define dynamics in several other ways using the defaults from the major notation programs as presets.

**Table 1:** A comparison of dynamic markings and corresponding MIDI velocities used by various notation programs.

**Musical dynamic**	**pppp**	**ppp**	**pp**	**p**	**mp**	**mf**	**f**	**ff**	**fff**	**ffff**
Finale MIDI velocities	10	23	36	49	62	75	88	101	114	127
MuseScore MIDI velocities		16	33	49	64	80	96	112	127	
Dorico default MIDI velocities^*^	5	14	25	46	61	77	89	101	119	123
Dorico linear curve MIDI velocities^*^	5	14	25	46	61	77	89	101	119	123
Sibelius MIDI velocities		16	39	61	71	84	98	113	127	

* *These results were achieved using the default curve set to 2.5 and a linear setting of 1*.

*Blacked-out areas represent dynamics not used by these programs*.

Using the minimum and maximum established amplitudes from the processes described above, *dyna's* representational default applies a logarithmic curve to the MIDI values between 0 and 127 taken from the Dorico notation program. Dorico's default dynamic scheme assigns these velocity values to the 10 most common dynamic values pppp, ppp, pp, p, mp, mf, f, ff, fff, and ffff. However, unlike the other leading notation programs, the developers of Dorico have wisely left room at the top of their dynamic range (velocities 124–127) to accommodate for what audio engineers might call headroom, but which the authors here define as the volume above the perceived pain threshold. By default, incoming amplitude levels are thus evaluated by *dyna* based on these definitions. If situations or preferences call for changes to these definitions, the presets for other dynamic schemas are available via the menu at the bottom right of *dyna's* user interface.

The user may also create unique dynamic presets (which are savable as Ableton Live.adg files for users of Max-for-Live). However, using the Dorico default has one interesting benefit in the case of the *dyna* program. Once the system has defined its context-dependent dynamics, the upper limit will remain stable unless the system suddenly encounters a greater dynamic. Thus, *dyna* can also be used as a pedagogical tool, as, a few minutes into a performance, greatly expanded dynamic peaks can be jarring to human listeners. In this case, a user employing *dyna* with the default settings would see the system register the “pain” dynamic before resetting the maximum, thus alerting the performer to a dynamic unevenness they might not have discovered otherwise. Overall, once the context-dependent dynamic system is trained, it will listen to the player intelligently and report on the player's dynamic performance, allowing the player to better assess their dynamic proficiency vs. what is on the written page.

## The *AvatarPlayer* Program

The *AvatarPlayer* program uses Markov note-to-note state transitions derived from a living composer–performer's improvised performances as a “choice engine,” providing a dynamically sensitive duet while listening to a live performance on the vibraphone. Using this system, the percussionist improvises on the vibraphone while the system listens, playing when he is playing and stopping when he stops. When the *AvatarPlayer* plays, it generates novel pitch content based on the Markov model of the player's style, filtered through several algorithmic AI processes. While the Markov model provides statistical probabilities to drive note choice, these algorithmic AI behavior processes change the way these data are used and are patterned after real-life vibraphone improvisation techniques used by the percussionist model.

**Figure 10:**
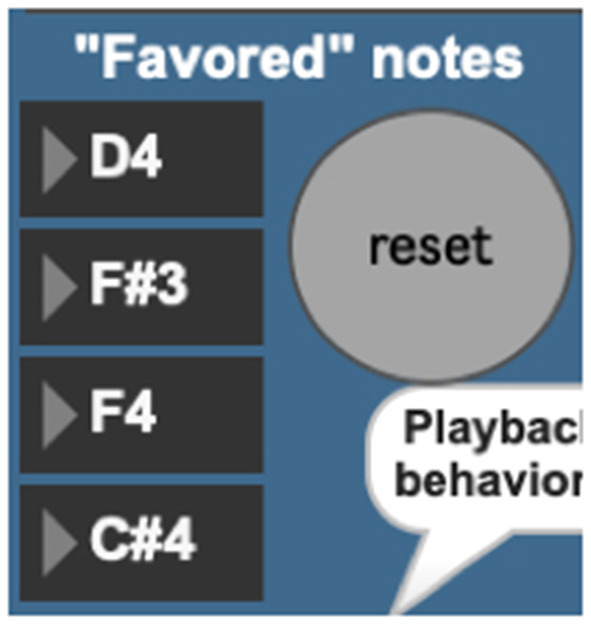
AvatarPlayer's four favored notes, chosen via a machine-learning database.

**Figure 11:**
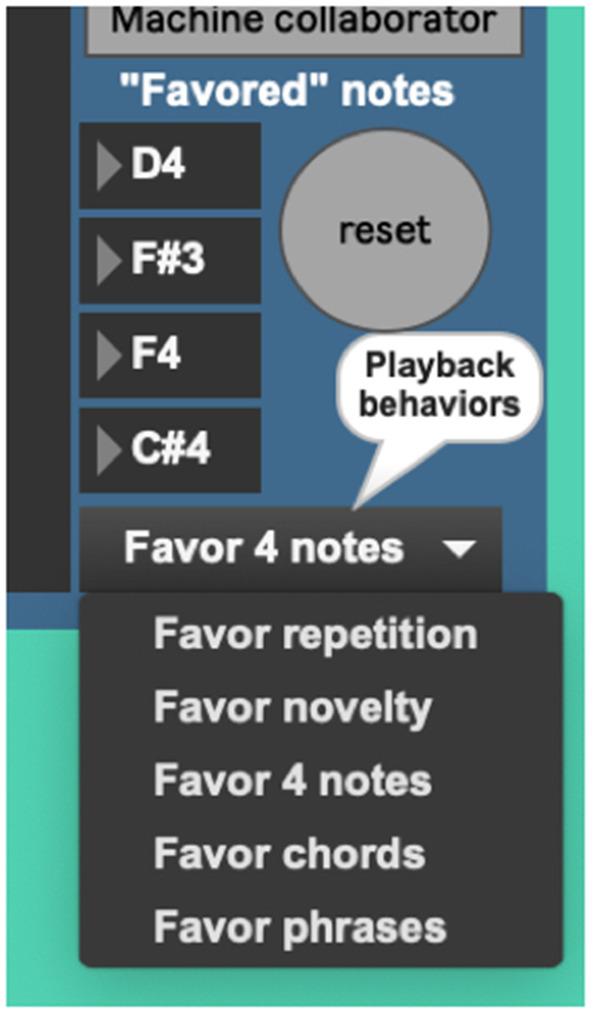
AvatarPlayer's various playback behaviors.

The *AvatarPlayer's* use of the Markov model is currently governed by five playback behaviors. The first such behavior (“favor four notes”) queries the Markov model for four notes to favor in its performance (Figure 10). Favoring these four chosen notes gives the note output a noticeable tonal centricity, a quality noticeably common in the live performer model's playing style. The behavior “Favor novelty” queries the Markov model for note-to-note transitions one by one, which creates a somewhat randomized, atonal quality. The behavior “Favor repetition” picks one note and favors repeating it two-out-of-three times. Cycling through these behavior modes (Figure 11) provides the system with a performance that sounds more human and less randomized, a common complaint against many generative music applications.

*AvatarPlayer* is also equipped with an autonomous AI mode, which makes musical accompaniment without listening to the live input. This mode is often useful in sound checking and system tests and can even be MIDI-mapped in Ableton Live so as to be turned on and off during performance. Its MIDI output can be easily recorded, so as to generate new compositional material, but *Avatar's* true purpose is as a collaborator using the sharp message from *sig2*~ and the dynamic velocities from the *dyna* program to create a sensitive context-dependent musical collaborator that actively listens as a musician would in a duet.

To further invoke a blended human-machine cyborg aesthetic, *Avatar's* developers have also created a number of high-quality Ableton Live instruments using the percussionist-model's own vibraphone as the sound source. In practice, the *Avatar* system listens to a human playing a vibraphone and plays along sensitively using an audible simulacrum of the vibraphone, behaving to a certain degree in the manner of the original human it is modeled after.

## Building the Machine Learning Model: The *AvatarMachineLoader* Program

**Figure 12:**
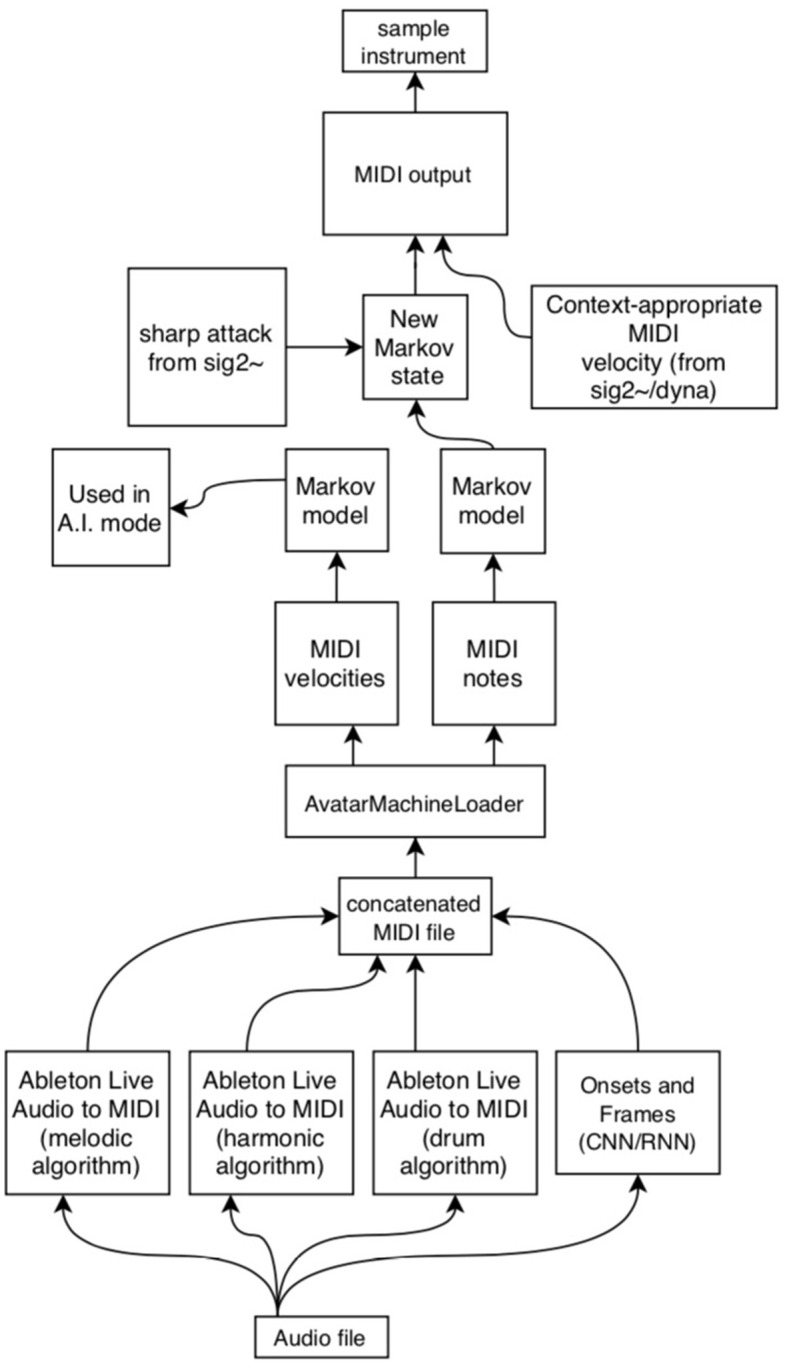
A schematic of how various machine learning technologies are used to create a Markov state transition table for a given performance.

As described above, the *AvatarPlayer* program makes use of a Markov model of pitch state transitions gathered from an analysis of real audio (Figure 12). The first hurdle in accomplishing this task is the transcription of a large number of audio files into MIDI files that the *AvatarMachineLoader* program may analyze. Though there are a number of systems designed to do this currently available to the user, the best and most easily obtainable is the *Onsets and Frames* model released by researchers at Google Magenta. This model may be used online quite easily or implemented via open-source JavaScript or Python code (Hawthorne et al., 2017). *Onsets and Frames* achieves this level of accuracy by using two separate neural networks (a convolutional neural network and a recurrent neural network) to detect pitch onsets even in polyphonically complex audio files.

As the *Onsets and Frames* model is designed at present to listen for piano timbres, rather than vibraphone, results are somewhat mixed. To improve accuracy of the eventual model, the authors also produced MIDI files using Ableton Live's built-in audio-to-MIDI transcription features, which use three different algorithms to assess pitch (focusing variously on melodic pitch movements, harmonic clusters, or rhythms). By painstakingly converting each audio recording using all four of these models, the authors hoped that the dataset (combined into a single multi-hour MIDI file) is significantly large enough to decrease the prominence of false positives and misapprehended pitches (Li et al., 2018). As these systems represent a sort of black box, it is difficult to say definitively whether the present model is an accurate representation of the original player. Accordingly, this procedure was repeatedly tested using MIDI files of music by gold standard composers like J.S. Bach and Vince Guaraldi. As new improvisations are supplied by the model composer–performer, the audio files are converted in these four ways and added to an ever-growing concatenated MIDI file.

Once the MIDI file dataset is compiled, the file must be passed through a Markov chain generator object (*ml.markov*) in order to produce a database of state transitions. The authors have achieved the best results setting this object to “order 4.” This setting produces state transitions that take into account the previous three notes. Since the ultimate goal is to create a model for vibraphone performance, the developers thought it best to generate a Markov model that took into account standard vibraphone performance technique, which utilizes four mallets in two hands, meaning the performer is often commonly improvising using groups of four notes.

While the present *AvatarPlayer* program utilizes a database of only pitch-to-pitch state transitions, the *AvatarMachineLoader* program also uses additional *ml.markov* objects to generate state transitions for MIDI velocities, durations, and harmonies. These late additions will eventually make it possible to generate novel material that is patterned after a more complete model of the original living composer-performer's performance. After these models are built, they may be saved as a simple text file that can be loaded into the *AvatarPlayer's* own *ml.markov* object. Once the *AvatarPlayer's* model is loaded and built, a simple *bang* message will generate new MIDI pitches conforming to the model's state transitions.

## Results

The *Avatar* system, consisting of *sig2*~, *dyna*, and the *AvatarPlayer*, was successfully debuted at the Fata Morgana music and art festival in Indianapolis on October 3, 2019, by percussionist and Professor Scott Deal. The system performed admirably and has since been featured at the MusicaAcoustica festival in Beijing, China, on October 22, 2019. These performances were well-received, and it has been reported that the system is rather easy to use and implement, even in the absence of the developers. A number of national and international performances for 2020 have already been scheduled.

It is often difficult to quantify the success or failure of musical experiments where the end result is a creative phenomenon. Such is the case with the present system in that the end result is not quantifiable data, but public performances using the system. As outlined above, the public performance record of this system is still in its infancy. While the recent results have all been promising, the pool of users will be extremely limited until such a time as the *Avatar* system is released commercially. As such, a commercial release date of February 28 has been announced, after which the system may be tested by the public and hard data may be collected and assessed.

A data-driven comparison of similarities between the living composer–performer upon which the system is modeled and the model itself would be a useful metric by which the system could be judged. The authors are presently beginning work on a future paper involving assessments of this type, which could be used as a model for assessments of future musical machine learning projects.

## Discussion, Scalability, and Limitations

Though various other technologies exist that purport to translate volume into MIDI velocity, results from this context-dependent system have been encouraging. The uses of more accurate instantaneous audio to MIDI transcription are many, as MIDI is the definitive control protocol underlying any music technology. It is hoped that in the near future, systems like Onsets and Frames may evolve into easily implementable real-time audio-to-MIDI. However, even with reliable real-time audio-to-MIDI, technologies will do little to cure the lack of context-based dynamics outlined above.

That said, the context-dependent dynamic system outlined above has many other potential applications. By filtering out excess noise and amplitude overages, this system could be adapted to control lighting and video effects, or to transcribe audio to dynamic notation in real time (perhaps as a plug-in for one of the notation programs mentioned above). The minimum and maximum definitions defined by *sig2*~ could be used to automatically (and cheaply) mix audio channels in situations where a professional audio engineer is unavailable. Systems like Landr, which use machine learning to mix or master audio files, are already affecting the market. Perhaps a context-dependent system for dynamics could do similar things for the ensemble classroom. But still, the system's most exciting possibilities revolve around creating new music by artificial intelligence or in enhancing the performance capabilities of human musicians with technology (Rowe, 1992; Miller, 2003; Weinberg and Driscoll, 2006).

While the present system works reliably, much more work is on the horizon. At the outset, the authors sketched out a goal of building a system that would build a machine learning model of what it hears in real time, save it, and update the model as more data became available. The present system does these things in achingly slow fashion, and not in real time. Another drawback of the lack of real-time adjustment has only become apparent after repeated use of the system. Users of the system have recently reported that since the present system does not save its definitions for the perceptual frameworks of silence and the pain threshold from session to session, *Avatar* seems to begin each session with a heightened sensitivity to loud sounds, and takes a significant amount of interaction with a player before the system is trained to react appropriately. It is hoped that along with the focus on making real-time machine learning models, the system will also eventually be able save these adaptations and remember them in future sessions. With the advent of very reliable audio-to-MIDI transfer via models like *Onsets and Frames*, the authors hope that it may be possible to make real-time machine learning a feature of the *Avatar* system in the near future.

The current focus on dynamics, while fundamental, presents only one important parameter of musical performance. To truly listen to music like a human, the system must also listen for, understand, and differentiate pitch, timbre, and duration. Beyond these low-level features, there are high-level features, like mood, emotion, tonal implications, and many others that provide much of the richness inherent in the best music of all genres. Adapting *Avatar* for durational perception, closely related to the concepts of dynamics and silence, has already begun in earnest. The machine learning tools currently extant within the Max environment (notably, the previously mentioned *ML*.^*^ and the *ml.lib* externals package from Nick Gillian), while brilliantly developed, leave much to be desired in the way of easy connections to common musical practice. Creating Markov models for monophonic pitch-to-pitch transitions are useful and simple to build at present but doing the same for a given MIDI file's harmonic content or articulative character requires a complete redesign of the system's inputs and outputs.

Another limitation of the project as it stands is the dependence on the *bonk*~ object's timbre recognition capabilities, which could be enhanced greatly. At the very least, adding multiple timbral models to *Avatar* will allow the user to use instruments other than the vibraphone, greatly widening the user base. Once these systems are improved, it should not be difficult to provide the system with timbral recognition capabilities. The ultimate goal of the authors is to provide a truly intuitive program that listens, rather than having to be managed by a knowledgeable user. These and the many other goals of this team will take much time and considerable hard work, but the rewards of such an enterprise are well-worth the effort.

## Other Information

Project Link: http://tavellab.net/

Operating system: Mac OSX 10+ / Windows 10+.

Programming language: Max standalone, also works as Max-for-Live device within Ableton Live.

Restrictions for non-academic use: none.

## Data Availability Statement

The data analyzed in this study is subject to the following licenses/restrictions: Creative Commons v 4.0, CC BY-SA (Attribution-ShareAlike). Requests to access these datasets should be directed to Jason Palamara/IUPUI (japalama@iu.edu).

## Author Contributions

JP wrote the manuscript and all code. WD provided feedback for the software, beta testing the software, and providing the original musical performances upon which the ML database was based.

## Conflict of Interest

The authors declare the following financial interest: this software has been commercially released for a nominal fee through http://tavellab.net/.
